# Macrophage migration inhibitory factor in acute kidneyinjury

**DOI:** 10.3389/fphys.2022.945827

**Published:** 2022-09-02

**Authors:** Yiwei Du, Hao Hao, Heng Ma, Hongbao Liu

**Affiliations:** ^1^ Department of Nephrology, Tangdu Hospital, Air Force Military Medical University (Fourth Military Medical University), Xi’an, China; ^2^ Department of Physiology and Pathophysiology, School of Basic Medical Sciences, Air Force Military Medical University (Fourth Military Medical University), Xi’an, China

**Keywords:** acute kidney injury, antioxidant, macrophage migration inhibitory factor, proinflammatory factor, renal tubular epithelial cell

## Abstract

Acute kidney injury (AKI) is a complex clinical syndrome with multiple etiologies and pathogenesis, which lacks early biomarkers and targeted therapy. Recently, macrophage migration inhibitory factor (MIF) family protein have received increasing attention owing to its pleiotropic protein molecule character in acute kidney injury, where it performed a dual role in the pathological process. macrophage migration inhibitory factor and macrophage migration inhibitory factor-2 are released into the peripheral circulation when Acute kidney injury occurs and interact with various cellular pathways. On the one hand, macrophage migration inhibitory factor exerts a protective effect in anti-oxidation and macrophage migration inhibitory factor-2 promotes cell proliferation and ameliorates renal fibrosis. On the other hand, macrophage migration inhibitory factor aggravates renal injury as an upstream inflammation factor. Herein, we provide an overview on the biological role and possible mechanisms of macrophage migration inhibitory factor and macrophage migration inhibitory factor-2 in the process of Acute kidney injury and the clinical application prospects of macrophage migration inhibitory factor family proteins as a potential therapeutic target.

## Introduction

Acute kidney injury (AKI) is a clinical syndrome characterized by rapid serum creatinine increase or urine output decrease or both within a short period of time (Bellomo et al., 2012; Ronco et al., 2019; Roy and Devarajan, 2020; Thongprayoon et al., 2020; Kellum et al., 2021), derived from cardiac surgery, liver transplantation, cisplatin, acute pancreatitis, and renal ischemia reperfusion (Gameiro et al., 2018; Griffin et al., 2019; Ronco et al., 2019; Yalcin et al., 2019; Bonavia et al., 2021; Kellum et al., 2021). Due to the diversity of etiology and the complexity of the pathophysiological mechanism, the existing diagnostic criteria like serum creatinine and urine volume are incapable of reflecting early kidney injury (Thomas et al., 2015; De Rosa et al., 2016; Griffin et al., 2019; Kellum et al., 2021). Up to now, there has been a lack of early sensitive biological markers and effective targeted drugs to ameliorate AKI in clinical practice (Gaiao and Paiva, 2017; Kellum and Prowle, 2018; Liu et al., 2020; Thongprayoon et al., 2020). Considered to be a heterogeneous clinical syndrome with various etiologies, pathogenesis and outcomes, AKI lacks targeted diagnosis and treatment (Kellum and Prowle, 2018; Vijayan, 2021). Accordingly, it is of great significance to pay attention to the common pathological mechanism of different etiologies and the related kidney-derived cytokine.

Macrophage migration inhibitory factor (MIF) is a soluble factor identified initially during the activation of T lymphocytes that inhibited random migration of macrophages (Weiser et al., 1989). MIF is a widely expressed pleiotropic cytokine, which has been subsequently confirmed to be produced by epithelial cells, endothelial cells, and endocrine cells in addition to macrophages (Kellum and Prowle, 2018; Jankauskas et al., 2019). A study has prompted new clues that show the role of MIF in AKI, indicating elevated urine MIF levels in patients were accompanied by the occurrence of AKI and urine MIF can be used as a potential biomarker of acute kidney injury in patients with acute pyelonephritis (Hong et al., 2012). Subsequent studies found the serum level of MIF increased in the early stage of AKI, and a multitude of experimental results have proved that the MIF family plays an essential role in the AKI model caused by different causes, which is a potential target for predicting and treating AKI (Pohl et al., 2016; Baron-Stefaniak et al., 2017; Stoppe et al., 2018). Therefore, it is necessary to further explore the role of MIF in the pathogenesis, so as to provide clues and ideas for the early diagnosis and subsequent treatment of AKI.

Focusing on the relationship between MIF and renal tubular cells, the pathological mechanism of MIF and AKI, and its application prospects in clinical treatment, this review will discuss the expression and role of MIF and its receptors in renal tubular cells, the specific signaling pathways through which MIF plays a role and the potential clinical diagnosis and application value of MIF.

### Basic biological functions of macrophage migration inhibitory factor

In 1989, Weiser et al. firstly reported that MIF was a 12.5KD protein molecule coded by activated T cell cDNA, consisting of 115 amino acids (Weiser et al., 1989). MIF exerts its biological activity in the form of homotrimers, with its sequence highly conserved in mammals, and it also exists in bacteria, nematodes, and protozoa. It can be pro-inflammatory cytokine (Tohyama et al., 2008; Lyu et al., 2021; Schindler et al., 2021; Vrataric et al., 2021), chemokine-like functional chemokine, anterior pituitary hormone, nuclease (Wang Y. et al., 2016), enzyme with tautomerism and thiol protein oxidoreductase (TPOR) activity (Stoppe et al., 2018), in parallel it can also regulate cell proliferation and survival, fibrosis (Sanchez-Nino et al., 2013) and energy metabolism (Gligorovska et al., 2021). Autocrine or paracrine MIF can bind to membrane surface receptor CD74/CD44 (Rice et al., 2003; Ma et al., 2010; Xie et al., 2016; Wang J. et al., 2021) and chemokine receptor CXCR2, CXCR4 and CXCR7 (Bernhagen et al., 2007; Chatterjee et al., 2014; Alampour-Rajabi et al., 2015) to jointly activate downstream signaling pathways such as ERK1/2, MAPK and P53. In the rat ischemia-reperfusion model, compared with the sham-operated group, it was observed that the expression of CD44 in renal tubular epithelial cells was significantly increased in the early stage of reperfusion in the surgical group (Kocak et al., 2009). MIF is also able to interact with JAB1/CSN5, NM23-H1, TXNIP and other intracellular proteins to affect other signal pathways (Jung et al., 2008; Wang L. et al., 2016; Kim et al., 2017; Jankauskas et al., 2019).

Currently, the MIF family includes MIF and MIF-2, known as D-dopachrome tautomerase (D-DT). Compared with MIF, MIF-2 lacks the LR domain necessary for CXCR2 activation, but they are very similar in structure and function. MIF-2 is also released when stimulated by LPS and can interact with CD74 receptor and JAB1 protein, showing that there is a large degree of synergy between them (Merk et al., 2011; Tilstam et al., 2021). Among those receptors of MIF, MIF-2 binds to CD74 with high affinity (Merk et al., 2011).

In addition, MIF is found expressed in a variety of cells, such as macrophages, epithelial cells, endothelial cells, and cardiomyocytes (Kellum and Prowle, 2018; Sumaiya et al., 2022). However, the distribution of MIF-2 was rarely known. Basal MIF expression is regulated by the transcription factor-specific protein1 (SP1) (Roger et al., 2007), cAMP response element binding protein (CREB) (Weiser et al., 1989)and NF-κB regulation (Chen et al., 2009). What’s more, ICBP90 (Yao et al., 2021)and HIF1-α can up-regulate the transcription level of MIF (Welford et al., 2006; Zis et al., 2015; Safi et al., 2020). Expressed in different tissues and cells, MIF acts different roles in many diseases, such as tumors (Du et al., 2013; Fukaya et al., 2016), autoimmune diseases (Hernandez-Palma et al., 2019) and acute organ injury (Djudjaj et al., 2017; Ochi et al., 2017) (Liu et al., 2021). The MIF protein is pre-formed and stored in the cytoplasmic vesicle structure. When exposed to hypoxia (Ma et al., 2010; Hofmann et al., 2021), lipopolysaccharide (LPS) (Yao et al., 2016; Toldi et al., 2021), tumor necrosis factor (TNF- α) (Cao et al., 2006)and other external stimuli, MIF is swiftly released into the peripheral blood circulation. It is worth mentioning that the release of MIF induced by hypoxia is biphasic. In the early stage of hypoxia, MIF is released in large quantities with the increase of hypoxia time, suggesting that it is derived from the pre-storage of MIF, and the secretion level decreases for a period of time and then increases again, suggesting that it may be *de novo* synthesis of hypoxia-induced MIF (Simons et al., 2011). Recent studies have demonstrated that MIF can be expressed in renal tubular epithelial cells (Djudjaj et al., 2017), especially in the tubules with ischemia-reperfusion injury, suggesting that there is a pathological link between MIF and AKI.

## Macrophage migration inhibitory factor and its receptor expression in kidney

**FIGURE 1:**
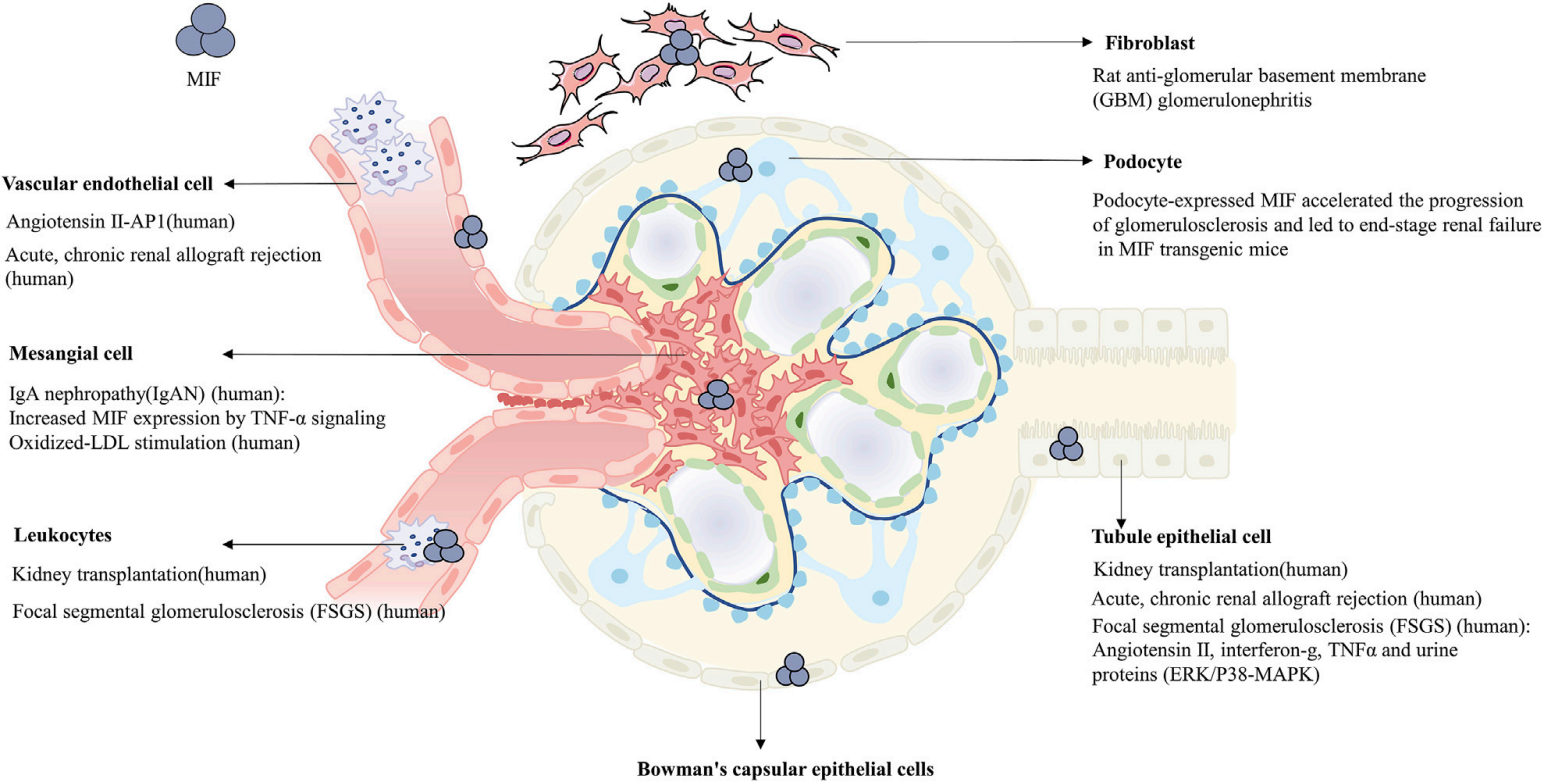
Expression of MIF in multiple cells of normal or pathological kidney, in which the diseases and concrete upregulated pathways participating in are noted respectively.

MIF is expressed in different cells of normal kidney (Figure 1), such as glomerular podocytes, mesangial cells, endothelial cells, renal tubular cells, fibroblasts, vascular smooth muscle cells and leukocytes (Rice et al., 2003; Sasaki et al., 2004; Sanchez-Nino et al., 2013; Musial and Zwolinska, 2021; Tziastoudi et al., 2021). MIF will be released and subsequently interacts with various pathways when cells are exposed to different damages. Previous studies have implicated that MIF in the injury response is predominantly derived from the synthesis and secretion of renal tubular epithelial cells (Kim et al., 2000; Lan et al., 2000; Rice et al., 2003; Stefaniak et al., 2015), which was further identified by bone marrow reconstruction and gene mouse hybridization in the study of Li et al. (Sumaiya et al., 2022), while the concrete proportion remains unknown compared with other sources of MIF. There are also two distinct conformational isoforms in immunology: oxidized MIF and reduced MIF, with oxidized MIF expressed in pathological state (Djudjaj et al., 2017).

CD74 is the cell surface receptor of MIF and its expression is influenced by MIF. In cisplatin-induced AKI and ischemia-reperfusion induced AKI models, the expression of CD74 was diminished in MIF knockout mice while significantly increased in both renal tubule cells and pro-inflammatory cells of wild-type mice. Western blot analysis revealed that MIF and CD74 expression levels were consistent, suggesting that MIF not only binds to CD74 but also there exists a regulatory relationship between them (Li et al., 2018). The binding of CD74 and MIF exerted protective effect after myocardial reperfusion (Miller et al., 2008), in a cohort of patients undergoing cardiac surgery, circulating sCD74 levels were distinctly reduced after a trend that was contrary to MIF and MIF-2 expression levels (Stoppe et al., 2015). Likewise, it was found that patients with detectable sCD74/MIF complexes in the serum had a significantly reduced incidence of postoperative acute kidney injury. Further, studies showed that the formation of sCD74/MIF complex significantly increased the redox activity of MIF, suggesting that sCD74 binding with MIF may enhance the TPOR activity of MIF, but the specific mechanism remained unclear (Stoppe et al., 2015). Compared with the treatment of rMIF alone, the combined action of sCD74 and rMIF effectively ameliorated oxidative stress and cell death in primary renal tubular epithelial cells cultured *in vitro* (Unruh et al., 2019). Although sCD74 *in vivo* treatment diminishes renal tubular cell damage, it exerts no apparent effect on blood creatinine. Hence, the specific source of sCD74 in the body, the effect of concentration and the specific molecular mechanism still need to be investigated (Stoppe et al., 2018). MIF can bind to CD74 to exert antioxidant effect, at the same time, it can also induce inflammation cascade via CD74-NF-κB (Li J. H. et al., 2019). Whereas, the underlying mechanism needs to be investigated more deeply.

In renal tissue, proximal tubule epithelial cells paly a material transport function in the physiological state and are susceptible to injuries like ischemia, hypoxia and nephrotoxicity (Kellum and Prowle, 2018). Single-cell RNA sequencing (scRNA-seq) indicated that there were different degrees of ferroptosis, necrosis, and pyroptosis in renal tubular epithelial cells after I/R injury (Zhao et al., 2020). Since the damage and repair of renal tubular epithelial cells have a crucial impact on the occurrence and development of diseases (Guo et al., 2019), the multiple effects of MIF as a pleiotropic molecule need to be well studied and delineated in renal tubular epithelial cells.

## Macrophage migration inhibitory factor in different models of acute kidney injury

AKI has caused great concern worldwide due to its high mortality rate and its predominant causes such as infections, hypovolemic shock, sepsis, drugs or invasive procedures (Kellum et al., 2021). Recently, it is observed that either plasma or urinary MIF level is elevated in AKI caused by liver transplantation (OLT) (Stefaniak et al., 2015), sepsis (Pohl et al., 2016; Li et al., 2022), uropathogenic *Escherichia coli* (UPEC) (Hong et al., 2013), cardiac surgery (Stoppe et al., 2018; Averdunk et al., 2020), cisplatin (Li et al., 2018; Zhang et al., 2020), acute pancreatitis in pregnancy (APIP) (Li M. et al., 2019; Liu et al., 2021), acute pyelonephritis (APN) (Hong et al., 2012), rhabdomyolysis (Nishida et al., 2015), or ischemic reperfusion injury (IRI) (Ochi et al., 2017; Li J. H. et al., 2019), on the other hand it is also observed that MIF is expressed in injured tubular epithelial cells remarkably, which denotes the closed relationship between MIF and AKI. In these studies mentioned above, MIF was found to be related to apoptosis, necroptosis, pyroptosis and ferroptosis of tubular epithelial cells. Intriguingly, in experiments, MIF can either rescue the cell death or aggravate the progression and ultimately be detrimental to the kidney. Likewise, the same conclusion can be drawn from clinical cases owing to the concentration of MIF and the outcome of patients of AKI. The expression, localization and effects of MIF in different models of AKI are summarized in Table 1.

**TABLE 1:** MIF and MIF-2 in different models of AKI.

Molecule	Disease	MIF localization	Species	Effect	Ref
MIF	Orthotopic liver transplantation (OLT)	Increased plasma MIF	Human	Harmful	Stefaniak et al. (2015)
	Sepsis	Increased plasma MIF, tubular cells	Human, mouse	Harmful	(Pohl et al., 2016; Li et al., 2022)
	Uropathogenic *Escherichia coli* (UPEC)	Increased urinary MIF, renal cortical tubules	Mouse	Unclear	Hong et al. (2013)
	Cardiac surgery	Increased plasma MIF, increased urinary MIF renal tubular epithelial cells	Human, mouse	Protective	Stoppe et al. (2018)
	Cisplatin	Increased plasma MIF, renal tubular epithelial cells	Mouse	Harmful	(Li et al., 2018; Zhang et al., 2020)
	Acute pancreatitis (AP)	Increase in fetal kidney tissues, kidney tissues	Rat, mouse	Harmful	(Li et al., 2019a; Liu et al., 2021)
	Acute pyelonephritis (APN)	Increased urinary MIF	Human	Unclear	Hong et al. (2012)
	Rhabdomyolysis	Increased plasma MIF	Mouse	Harmful	Nishida et al. (2015)
	Ischemic reperfusion injury (IRI)	Increased plasma MIF, increased urinary MIF	Human, mouse	Harmful	Li et al. (2019b)
MIF-2/D-DT	Orthotopic liver transplantation (OLT)	Increased plasma MIF	Human	No association with AKI	(Baron-Stefaniak et al., 2019)
	Ischemic reperfusion injury (IRI)	Not mentioned	Mouse	Protective	(Ochi et al., 2017)

Among the ischemic reperfusion injury models, even the difference of clamping unilateral or bilateral renal artery can also contribute to distinctively inverse results on account of the concentration of MIF. It is speculated that high concentration of MIF is more likely to cause inflammation, as shown in bilateral IRI-AKI and cisplatin-induce AKI, where the average level of serum MIF concentration is higher than 1,500 ng/ml and apparent inflammation could be seen (Li et al., 2018; Li M. et al., 2019). Conversely, lower MIF concentration in AKI patients after cardiac surgery tends to exert protective effects on damage to kidney (Stoppe et al., 2018).

These opposing results prompt us to consider when high levels of MIF occurred in AKI, whether during the compensatory or decompensated phase, which has not been adequately studied. Although different inducers all lead to AKI, we need to be aware of each kind of AKI possessing its unique pathophysiology and different emphasis on damage. Maybe it is the exact reason to explain a distinctive role of MIF in different models. Though the level of MIF was upregulated, the underlying mechanism and concrete pathways involving MIF need to be discussed and lucubrated furthermore. More clinical cases and experiments are also needed to confirm those hypotheses.

## Pathophysiological role of macrophage migration inhibitory factor and macrophage migration inhibitory factor-2 in acute kidney injury

Acute kidney injury is caused by hypoxia and oxidative stress due to impaired microcirculation and unbalanced energy demand, which subsequently causes inflammation infiltration, mitochondrial dysfunction, oxidative stress, and lipid peroxidation cell damage (Virzi et al., 2018; Gonsalez et al., 2019). It is worth noting that acute kidney injury caused by different etiologies all involves inflammation, oxidative stress, and damage to renal tubular epithelial cells by hypoxia and nephrotoxic substances (Kellum and Prowle, 2018; Peerapornratana et al., 2019). Therefore, interfering with common pathophysiological mechanisms through targeted cytokines is the key to solving these problems.

**FIGURE 2:**
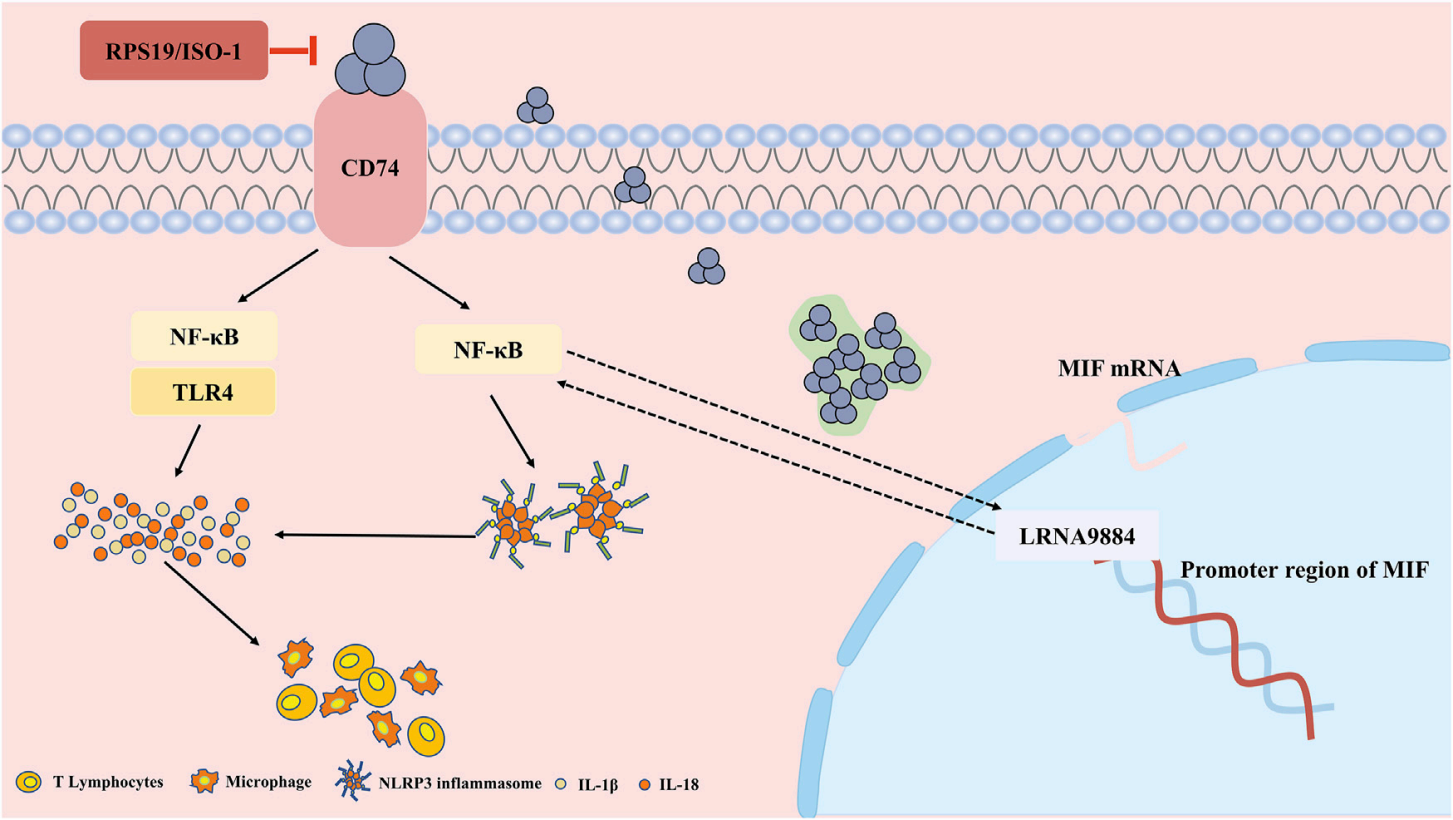
The pathophysiological pro-inflammation role and mechanism of MIF in acute kidney injury (AKI). In renal tubular epithelial cells, MIF binds to CD74 to induce the inflammatory cascade through NF-κB and NLRP3, while RPS19 or ISO-1 can ameliorate the injury. Besides, MIF is also regulated by long non coding RNA LRNA9884, binding to the promoter of MIF, further aggravates the inflammation infiltration.

In multiple clinical observational studies, circulating MIF level is elevated in early AKI patients, which is positively or negatively correlated with the severity of pathological damage (Bruchfeld et al., 2016; Pohl et al., 2016; Stoppe et al., 2018; Li J. H. et al., 2019). This might be due to differences in the number of clinical samples, observation time, and model differences. Besides, these facts unravel that MIF plays a role in the occurrence and development of AKI. The pleiotropic effect of MIF is embodied in not only a pro-inflammatory factor, but also an antioxidant and promoting cell proliferation. It is speculated that MIF may play a dual role in the pathological process of AKI. Figure 2

### Macrophage migration inhibitory factor and inflammation of acute kidney injury

Inflammation is a complex reaction of our body in order to eliminate pathogenic substances and repair damage. The balance between inflammatory factors and anti-inflammatory factors can assure that the body removes harmful substances to retain body homeostasis. Nevertheless, excessive inflammation, long-term hypoxia, and constant secretion of pro-fibrotic cytokines frequently result in abnormal repair processes, leading to post-AKI fibrosis and chronic renal insufficiency (Sanchez-Nino et al., 2013; Wang S. et al., 2016; He et al., 2017).

Known as a pro-inflammation factor, MIF was reported as a pathogenic role in AKI and might be a potential therapeutic target in the future. Recently, research has confirmed the role of MIF as an upstream molecule in the inflammatory cascade (Figure 2). MIF mediates NLRP3’s interaction with vimentin to activate and assemble inflammasome, and inhibition of MIF decreases secretion of IL-1β and IL-18 dependent on NLRP3 (Sanchez-Nino et al., 2013). NLRP3 inflammasome is a common pathway in inflammatory diseases, composed of sensors (NLRP3), adaptors (ASC) and effectors (caspase-1), and plays a role in the pathogenesis of acute inflammation of the kidney and tissue remodeling (Schroder et al., 2010). Elevated levels of NLRP3, caspase-1 and IL-1β protein expression and IL-1β and IL-18 transcription were identified in l-arginine-induced severe acute pancreatitis (SAP) (Liu et al., 2021), cisplatin (Li et al., 2018)and contrast agent related AKI (Xu et al., 2020)mouse models. After treatment with the MIF inhibitor ISO-1, the tissue pathological damage and inflammatory infiltration were reduced in the SAP-AKI model, along with decreased MIF mRNA level and NLRP3 expression level (Liu et al., 2021). However, the mechanism of interaction between MIF and NLRP3 in renal experiments is not well understood, and the application of ISO inhibitors is limited to animal experiments, lacking clinical practice validation.

Besides, another study has elucidated that MIF can induce renal inflammatory infiltration through CD74/NF-κB pathway. Deletion of MIF inhibited the expression of CD74, TLR4 (Toll-Like Receptor 4) and activation of NF-κB, with subsequently blood creatinine and renal tubular necrosis decreased after ischemia-reperfusion (Li M. et al., 2019). On the contrary, the MIF level in plasma and urine increased rapidly after IRI-AKI in WT mice, which was correlated with the increase in serum creatinine and the severity of tubular necrosis. Similarly, Li et al. (Li et al., 2018) found that in the cisplatin-induced AKI model, the deletion of MIF caused by gene knockout or drug RPS19 inhibition both improved kidney function, reduced tissue damage, and inhibited IL-1, IL-8 and inflammatory infiltration via inhibiting CD74-NF-κB. Furthermore, the inflammation of MIF could also be regulated by long non-coding RNA (Zhang et al., 2020). LRNA9884 directly bound to the promoter region of MIF, improving the transcriptional level of MIF, further promoted the renal inflammatory cytokine storm after IRI-AKI.

### Macrophage migration inhibitory factor and oxidative stress of acute kidney injury

Oxidative stress refers to the increase of reactive oxygen species (ROS) and reactive nitrogen (RNS). Oxidative stress in the kidney mainly derives from NADPH oxidase and mitochondria in the vascular system and kidney tissue. Low-level oxidative stress is dispensable for regulating cell survival, proliferation and other signals, whereas excessive oxidative stress can contribute to cell death and inflammatory infiltration, which is not conducive to tissue repair. In view of this, reducing oxidative stress is a necessary treatment strategy to prevent the occurrence and development of acute kidney injury. However, the cellular diversity and complexity of oxidative stress sources make current antioxidants fail to achieve good renal protection (Ratliff et al., 2016).

**FIGURE 3:**
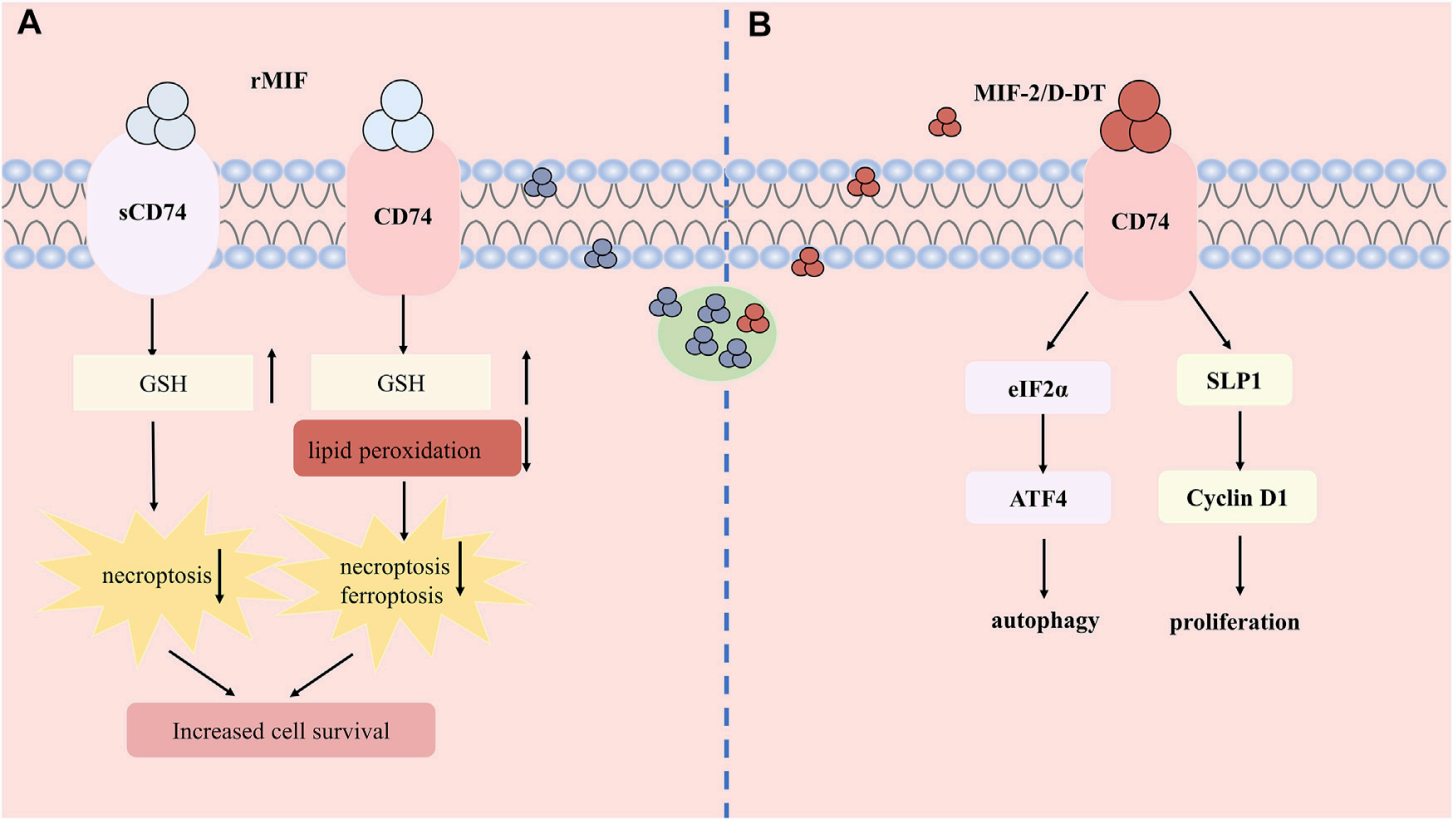
The protective role and mechanism of MIF and MIF-2 in acute kidney injury. **(A)** Recombined human MIF treatment can bind to CD74 or soluble form of CD74 (sCD74) to increase the content of GSH, reduce lipid peroxidation and attenuates necroptosis and ferroptosis. **(B)** MIF-2 can also bind CD74 to induce autophagy and promote cell proliferation.

Unlike the pathogenic role of pro-inflammation, current studies have shown that MIF may exert intracellular thiol - protein oxidoreductase (TPOR) activity (Kers et al., 2016)and reduce oxidative stress to protect the kidney from injury (Figure 3A). The CXXC motif in the center of the MIF molecule mediates TPOR activity, which usually exists in the thioredoxin (Trx) superfamily of TPORs (Thiele and Bernhagen, 2005). In the research of Christian et al. (Stoppe et al., 2018), experiments found that rMIF treatment *in vivo* and *in vitro* can increase glutathione and reduce lipid peroxidation, thereby reducing necrosis, ferroptosis, and inflammatory infiltration of renal tubular epithelial cells. In addition, sCD74 receptor ectodomain or combined with rMIF reduced tubular injury and necroptosis in the cortex, restoring the content of GSH. Meanwhile, the comparison of serum MIF detection in patients with AKI after cardiac surgery indicated that patients with high serum MIF showed higher total antioxidant capacity. The risk of AKI in patients with 12- hour high circulatory MIF is significantly reduced. S- nitrification in the early stage of myocardial ischemia-reperfusion can increase the accumulation of intracellular MIF, promote intracellular anti-oxidation and reduce the extracellular signal transduction of MIF. Except that, MIF can interact with thiol-specific antioxidant protein (PAG) (Jung et al., 2001) and inhibit its antioxidant activity, which can also act as a molecular chaperone to inhibit mutant superoxide dismutase (SOD1) misfolding and membrane binding (Israelson et al., 2015). However, the actual mechanism that how MIF exerting its antioxidant activity needs to be explored further.

### Macrophage migration inhibitory factor and fibrosis after acute kidney injury

Inadaptable damage repair after AKI can give rise to renal tubular cell cycle arrest in G2/M phase (Ferenbach and Bonventre, 2015; Kellum and Chawla, 2016; Gaiao and Paiva, 2017), and the damaged proximal epithelial cells can drive renal fibrosis through paracrine pro-proliferation and pro-fibrosis factors, thus helping cells overcome G2/M checkpoint is an effective treatment target (Bonventre, 2014; Canaud and Bonventre, 2015). It is reported that MIF exerted antifibrotic effects through CD74/AMPK pathway to resist liver fibrosis (Heinrichs et al., 2011). MIF can promote the proliferation of different cell types under pathological conditions in kidney (Chen et al., 2015; Safi et al., 2020; Wang Y. et al., 2021; Cao et al., 2021). For example, the up-regulation of the MIF-CD74/CD44 axis has been found to be associated with pathological glomerular cell proliferation (Djudjaj et al., 2016).

As we mentioned above, MIF exerts a renal protective role by regulating cell apoptosis, promoting cell proliferation and inhibiting fibrosis during the repair process after acute kidney injury. Immunofluorescence staining of kidney tissues of healthy people and fibrosis patients, control group and two progressive nephropathy model mice treated with IR or FA identified the expression of MIF in renal tubular cells decreased under pathological conditions. In mice with unilateral ureteral obstruction, gene deletion or pharmacological inhibition of MIF aggravates fibrosis and inflammation, even after fibrosis has occurred, treatment with recombinant MIF can still provide protection (Djudjaj et al., 2017).

### Macrophage migration inhibitory factor-2 and cell death and proliferation of acute kidney injury

MIF-2 was also found to show reno-protective effect on IR-AKI, via regulating apoptosis, autophagy and cell proliferation (Figure 3B). A study shows that MIF-2 treatment significantly improves hypoxia-induced renal tubular epithelial cell damage, and regulates the proliferation of surviving renal tubular cells by stimulating the up-regulation of secreted leukocyte protease inhibitor (SLPI) -dependent cyclin D1 expression, which activates the eIF2 *α* -ATF4 pathway to induce autophagy at the same time, illustrating that MIF-2 can also play a protective role in regulating autophagy and promoting cell proliferation, which can be used for early treatment of inhibiting AKI renal tubular cell death and differentiation (Shachar, 2017; Gameiro et al., 2018).

Studies have reported that different concentrations of MIF have different effects. High concentrations can stimulate inflammation, while low concentrations can promote cell proliferation (Cui et al., 2016; Marin et al., 2017). MIF itself is a pleiotropic molecule, therefore, with the passage of time, MIF may exert TPOR antioxidant activity in the early stage, promote cell proliferation and reduce fibrosis in the late stage. As for whether the inflammatory or protective effect is dominant, according to current studies, it may depend on the degree of injury, the concentration of MIF in the individual, and gender. Whereas studies about MIF-2 still remain rare and more investigation is needed.

## The value of macrophage migration inhibitory factor in the clinical diagnosis and application of acute kidney injury

A recent report suggested that the extended repeat CATT_7_ allele was associated with a higher risk of AKI, high serum MIF before cardiac surgery and death after surgery (Du et al., 2020). With more and more research and clinical data testing, MIF shows great potential in AKI being an independent predictive factor and is more advantageous than existing biomarkers owing to the fact that the source of MIF is from the kidney itself, acting pleiotropic and reacting to injuries quickly, which meets the criteria as a biological marker of AKI.

### Biomarkers

Traditional criteria such as urine volume and serum creatinine lack in specificity and sensitivity, and are unable to reflect the true injury of the kidney timely and accurately. Numerous clinical research studies indicated MIF showed a potential role in the prediction of the occurrence, aggravation or outcomes of diseases, which would facilitate the management of patients. Based on previous studies, MIF shows great potential in specificity and sensitivity. On the one hand, serum MIF is effective in distinguishing AKI and non-AKI patients (Baron-Stefaniak et al., 2017; Sumaiya et al., 2022). More elevated level of serum MIF concentration of the AKI group could be observed in the onset or early stages of the disease, other than in the non-AKI group. The same is true of the urinary MIF level in APN Patients (Hong et al., 2012). On the other hand, compared with serum cystatin c (Cys-c), interleukin 6 (IL-6), and procalcitonin (PCT), serum MIF levels increased with the development of AKI, suggesting its close association with different stages of septic AKI. Compared with sCr, serum MIF exerted a better predictive value for the requirement of renal replacement therapy (Stefaniak et al., 2015). The level of serum MIF at the same time also corresponded to the outcomes of AKI (Stefaniak et al., 2015; Stoppe et al., 2018). High serum MIF levels were associated with a reduced incidence of AKI after cardiac surgery, but with an increased incidence in septic AKI (Li et al., 2022). Collectively, MIF is a promising biomarker that could be applied in clinical practice with more external validation.

MIF-2 was reported to be elevated in critically ill people, associated with the parameters of organ damage (Pohl et al., 2017). However, the concentration of MIF-2 was not influenced by renal injury, which is an obvious difference from MIF. Since the studies concerning MIF-2 were scarce, the reliability of MIF-2 as a biomarker of AKI stills needs to be verified further.

### Macrophage migration inhibitory factor antagonist

In light of the pro-inflammatory effect of MIF as an upstream molecule in the inflammatory signaling pathway, small molecules aimed to negatively regulate MIF emerged. ISO-1 [(S,R)-3-(4-hydroxyphenyl)-4,5-dihydro-5-isoxazole acetic acid methyl ester] was identified as an effective inhibitor binding to the tautomerase active site of MIF (Al-Abed et al., 2005). What’s more, the application of ISO-1 was reported to reduce the mRNA and protein expression of MIF (Lv et al., 2013; Li J. H. et al., 2019). Multiple basic experiments indicated that ISO-1 inhibited the activation of P38MAPK (mitogen-activated protein kinase), NF-κB and NLRP3 pathway and further reduced inflammation response, exerting protective effect on AKI associated with SAP, APIP and shock (Li M. et al., 2019; Liu et al., 2021; Patel et al., 2022). And ribosomal protein S19 (RPS19) is an endogenous binding partner of MIF, further inhibiting its binding with receptor CD74 and CXCR2 (Filip et al., 2009). Similar to ISO-1, the use of RPS19 also blocked the increase of MIF and CD74 and the activation of ERK (extracellular-signal-regulated kinase) and NF-κB (Lv et al., 2013). These all might be related with MIF-CD74 pathway that can activate MAPKs, ERK1 and NF-κB, which subsequently initiated inflammatory cascade (Su et al., 2017).

In basic research, ISO-1 and RPS19 are regarded as potent anti-inflammatory agent, inhibiting the pro-inflammatory effect of MIF and exerting reno-protective effects in mice. However it has not been used in clinical trials since its safety, administration time, dosage and toxicity are still unclear.

### Macrophage migration inhibitory factor agonist

As mentioned before, there are also studies reporting that MIF plays an anti-oxidant and anti-fibrosis effect in AKI. The loss or decline of MIF in the pathological process makes cell death and tissue damage aggravated. For this effect, supplementing exogenous recombinant MIF (rMIF) or MIF agonists to enhance the MIF effect is a reno-protective strategy. MIF20, MIF21, and MIF33 were synthesized before and they were considered to induce a conformational change in MIF by increasing its receptor interaction and signaling efficiency (Wang et al., 2013). MIF-2 treatment can remarkably improve the restoration of damaged tubular cells, promoting regeneration (Ochi et al., 2017); scD74/rMIF was co-incubated with hypoxia-treated renal tubular cells and rMIF’s antioxidant effect was enhanced (Stoppe et al., 2018). MIF agonists were confirmed to enhance the phosphorylation of ERK1/2 in human fibroblasts dependent on CD74 (Jorgensen et al., 2010). In addition, MIF20 exerts a myocardial protective effect via MIF-AMPK pathway and subsequent myocardial glucose uptake in the ischemic myocardial injury model, however, its role has not been reported in acute kidney injury (Wang et al., 2013; Ma et al., 2020).

## Summaries and perspectives

The MIF family can not only play a protective role in anti-oxidation, promoting proliferation and reducing fibrosis in AKI, but also can damage kidney function as an upstream inflammatory pathway. This dual effect has been confirmed in clinical sample observation studies and basic experimental results. Inhibitors or enhancers produced by the existing MIF research mechanism have also played a corresponding role. However, as MIF is expressed in multiple tissues and cells *in vivo* and elderly patients have poor tolerance to drugs, we are faced with problems such as lack of tissue targeting, drug concentration, half-life and uncertain safety. Acute kidney injury is divided into different stages based on serum creatinine levels, with the role of MIF in each stage unclear. What’s more, the correlation between changes in MIF concentration, cell origin, changes in MIF concentration and creatinine concentration in each stage are all remaining enigmatic, where a large number of clinical samples are needed for further observation. The specific role and mechanism pathways of MIF, its structural homologue MIF-2 and surface receptor CD74 in the progression of acute kidney injury needs to be investigated deeply, especially the related research of MIF-2 is still relatively few. The clarification of these pathways helps to understand the dual role of MIF in AKI, which has important clinical significance for the early prevention of aged AKI and the inhibition of disease progression.
